# Alpha-Actinin-3 Deficiency Might Affect Recovery from Non-Contact Muscle Injuries: Preliminary Findings in a Top-Level Soccer Team

**DOI:** 10.3390/genes12050769

**Published:** 2021-05-18

**Authors:** Gil Rodas, Víctor Moreno-Pérez, Juan Del Coso, Daniel Florit, Lourdes Osaba, Alejandro Lucia

**Affiliations:** 1 Medical Department, Futbol Club Barcelona, Barça Innovation Hub, 08028 Barcelona, Spain; gil.rodas@fcbarcelona.cat (G.R.); daniel.florit@fcbarcelona.cat (D.F.); 2 Center for Translational Research in Physiotherapy, Department of Pathology and Surgery, Miguel Hernandez University of Elche, 03202 Elche, Spain; vmoreno@umh.es; 3 Centre for Sport Studies, Rey Juan Carlos University, 28943 Fuenlabrada, Spain; 4 Progenika Biopharma, A Grifols Company, 48160 Derio, Spain; lourdes.osaba@progenika.grifols.com; 5 Faculty of Sport Sciences, Universidad Europea de Madrid, 28670 Villaviciosa de Odón, Spain; 6 Research Institute Imas12, Hospital 12 de Octubre, 28041 Madrid, Spain

**Keywords:** football, genetic, muscle strain, sports injury, elite athlete

## Abstract

There are recent data suggesting an association between the R577X polymorphism (rs1815739) in the gene encoding α-actinin-3 (*ACTN3*) and the risk of musculoskeletal injuries. The purpose of this study was to analyze the association of rs1815739 with risk of, and recovery time from non-contact soft-tissue muscle injuries in professional soccer players. Forty-six (22 male and 24 female) players from a top-level professional soccer team were assessed during five consecutive seasons: the genotype distribution was: RR, 41.3%; RX, 47.8%; and XX, 10.9%. There was a trend towards a higher risk of muscle injury associated with the XX genotype (*p* = 0.092, with no injury-free XX player during the 5-year study period) and a significant genotype effect for the time needed to return to play (*p* = 0.044, with the highest value shown for the XX genotype, i.e., 36 ± 26 days, vs. 20 ± 10 and 17 ± 12 days for RR and RX, respectively). In conclusion, the XX genotype might be associated not only with a higher risk of non-contact muscle injuries, but also of recovery time from these conditions. However, more research in larger cohorts is needed to confirm this preliminary hypothesis.

## 1. Introduction

Soccer is a power-driven, ‘explosive’ sport where players are required to perform sprints, sudden accelerations and decelerations, rapid changes of direction and, in many situations, kicking the ball [1,2]. Accordingly, competitive soccer practice is associated with a high incidence of muscle injuries (e.g., two per player and season in Union of European Football Association (UEFA) teams [3]). The resulting time-loss from competition of important players poses a major problem for professional teams, both in terms of potential decrements in competition performance and financial costs [4].

Several non-modifiable factors such as age, previous injuries, muscle architecture and ethnicity [5,6] and modifiable factors such as flexibility [7], fatigue [3] or muscle weakness [5,8,9,10] can affect the risk of non-contact muscle injuries. Some gene polymorphisms might also be involved [11]. Particularly, the stop-codon variant p.R577X (c.1858C > T, rs1815739) in *ACTN3*, a skeletal muscle structural gene encoding α-actinin-3, is receiving growing interest [12]. α-actinin-3 is a major component of the contractile apparatus at the Z-line that is expressed almost exclusively in fast-twitch, type II muscle fibers [12,13], where it is needed to generate ‘explosive’ contractions and also plays a mechanical role by stabilizing actin thin filaments at the Z-line during muscle contractions (as reviewed in [12]). Individuals with the ‘null’ 577XX (or simply ‘XX’) genotype are unable to express α-actinin-3, as opposed to their RR or RX counterparts [12]. 

Recent studies conducted in soccer players have identified an association between α-actinin-3 deficiency and susceptibility to develop musculoskeletal injuries (i.e., two to threefold higher risk in XX than in RR individuals) [14,15]. Similar findings have been reported in marathoners [16], although other investigations have failed to find an association between the *ACTN3* R577X genotype and risk of muscle injury [17,18]. There is also evidence that XX individuals are more prone to high levels of exercise-induced muscle damage after a marathon [19] and a half-ironman triathlon [20], with α-actinin-3 potentially playing a protective role against muscle damage after eccentric training and improving stress-sensor signalling involved in tissue repair [21].

An important issue in the context of professional sports is not only predicting the risk of injuries, but also to accelerate the time to return-to-play once injury has occurred. In this context, there is some rationale to support that eccentric contractions or high-power movements would cause more strain on the muscle fibers of XX individuals than in those with the other *ACTN3* R577X genotypes [22], given the protective mechanical role played by this protein as a scaffold at the sarcomeric Z line [23]. Yet, recent research has failed to demonstrate a significant difference in the pre- vs. post-game increase in muscle damage markers across R577X genotypes in very young soccer players [24] or a significant association of *ACTN3* R577X polymorphism with time to recovery from non-contact soft-tissue muscle injury in professional players [15].

We aimed to determine the association of *ACTN3* R577X polymorphism with the incidence of non-contact (‘indirect’) soft-tissue muscle injuries and with the time needed to return to competition after injury onset in male and female professional soccer players.

## 2. Materials and Methods

The study protocol conformed to the Declaration of Helsinki for Human Research and was approved by the local research ethics committee (Consell Català del’Esport, Barcelona, Generalitat de Catalunya 01/2018CEICGC). Written informed consent was obtained from all participants.

The study was conducted solely with those players of the first division of the Spanish National League in both men’s (*LaLiga Santander*) and women’s (*Primera Iberdrola*) categories who played on the same team during five consecutive seasons (from July 2015 to June 2020). Thus, during this period, the same medical staff recorded all the injuries and the same medical and physiotherapy team supervised standard protocols for muscle injury recovery. The occurrence of muscle injury was ascertained with ultrasound or magnetic resonance imaging scans. Only muscle-type (‘soft-tissue’) injuries resulting from soccer training sessions or competitions and located in the lower limbs were recorded. Traumatic muscle injuries caused by contact with another player or produced during tackle actions were discarded from the analysis. Recorded data were introduced into a validated electronic medical record software (COR, FCB, Barcelona, Spain). All non-contact soft-tissue muscle injuries were diagnosed, classified and recorded using the classification system developed by the club’s medical staff for muscle injuries, following international guidelines [25,26]. The time to return to play was calculated as the time (in days) from injury until the player returned safely to training or competition, as detailed in the FC Barcelona Guide [27].

Blood samples from each participant were collected into EDTA vacutainer tubes. Genomic DNA was isolated using the QIAamp DNA Blood Mini kit (Qiagen, Hilden, Germany) following provider instructions and genotyping of the *ACTN3* rs1815739 polymorphism was performed using Kompetitive Allele Specific PCR (KASP™, Hoddesdon, UK) genotyping technology. Appropriate negative controls were included.

### Statistical Analysis

We determined whether genotype distribution met the Hardy–Weinberg Equilibrium (HWE). Descriptive statistics were calculated for each genotype. Differences among genotypes were calculated with the X^2^ test for those variables presented as frequencies and with one-way analysis of variance for continuous variables (with the Tukey test applied post hoc for pairwise comparisons). All statistical analyses were performed with statistical software (SPSS Statistics 22, IBM, Chicago, IL, USA). Descriptive data are presented as means and standard deviations. The level of significance was set at 0.05.

## 3. Results

Forty-six professional soccer players participated in the study (mean age 26.1 ± [SD] 4.6 years, 22 males and 24 females). The population sample included the best soccer player in the world according to *Fédération Internationale de Football Association* (FIFA). In the male team, there were several winners of the FIFA world cup and of major UEFA tournaments, whereas the female team had won the *Primera Iberdrola* competition twice and reached the semi-finals of the Women’s Champions League within the seasons under investigation. During the study period, the players trained for 6 and 33–35 weeks during the preseason and competition season, respectively (or 14–16 h/week and 8–10 h/week). A total of 96 muscles injuries were recorded in the sample of soccer players during the period of investigation.

Genotype success was 100%, with the following genotype distribution (which followed HWE): RR, 41.3%; RX, 47.8%; and XX, 10.9%. There were no differences in demographic characteristics across genotypes (Table 1).

**Figure 1:**
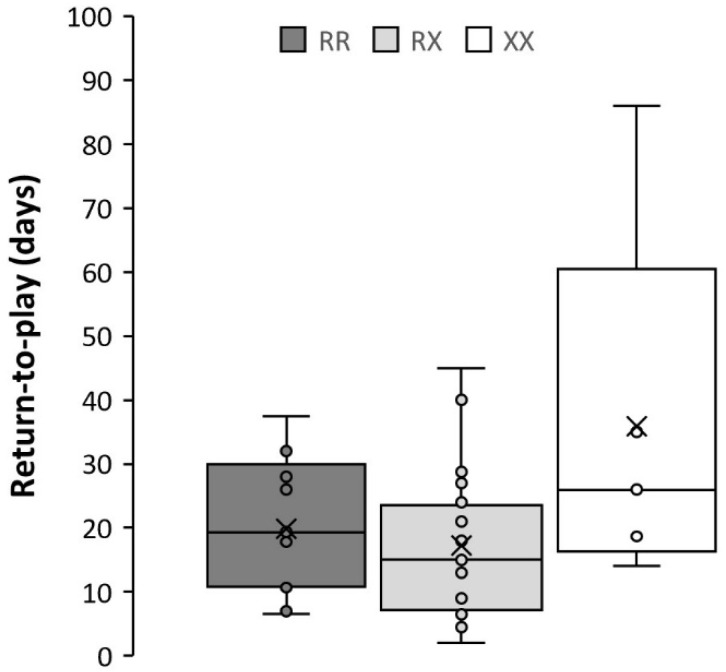
Box-and-whisker plots for the number of days from muscle injury to return-to-play in professional soccer players according to α-actinin-3 (*ACTN3*) R577X genotype. In each box, the bottom line represents the first quartile, the line within the box is the median and the upper line is the third quartile, and the whiskers are 1.5× interquartile range.

A trend towards a higher risk of injuries associated with the XX genotype (*p* = 0.092) was found, with no injury-free XX player during the 5-year study period (Table 2). In fact, the proportion of participants showing 1+ injuries was higher in the participants with the X-allele (RX + XX) than in their RR referents (*p* = 0.033). Nevertheless, among those players with 1+ injuries, the R777X genotype was not associated with the number of injuries (*p* = 0.799). On the other hand, there was a significant genotype effect for the time needed to return to play (*p* = 0.044), with the highest value shown for the XX genotype (i.e., >1 month vs. clearly below 30 days for RR or RX; Table 2 and Figure 1).

## 4. Discussion

Although preliminary in essence, our findings suggest an association between the null XX genotype—or the X-allele—and the risk of muscle injuries and especially time to return-to-play once muscle injury has occurred. Our results are overall in agreement with previous research suggesting that XX genotype might be the ‘risk’ genotype with regard to muscle injuries in soccer [14,15] and endurance running [16]. In addition, a novelty compared to previous research is the finding that the XX genotype seems to be associated with a longer recovery time. To the best of our knowledge, only a recent study conducted in the same soccer team but with different male players in essentially different (nonconsecutive) seasons from those studied here and including no female players [15], has previously addressed this issue.

Our results corroborate the notion that soccer is essentially an ‘explosive’ sport where high mechanical stress is imposed in lower-limb muscles as reflected by the lower proportion of the ‘slow’ XX genotype (i.e., 11% here) compared to the general population (~18% among Caucasians), which is in line with previous research in world-class soccer players (i.e., 7% in Clos et al. [15], and 11% in Santiago et al. [28]). The physiological basis for a link between α-actinin-3 deficiency (compared to RR or RX genotypes) and susceptibility to non-contact muscle injury is generally attributed to alterations in the force transmission structures of the myofilaments at the level of the Z-disc [29]. As α-actinin-3 is indeed a protein with a key role in anchoring actinin filaments to the Z-disc, α-actinin-3 deficiency might lead to a muscle phenotype with a lower capacity to tolerate the strain produced by ‘explosive’ sports actions [12].

The time needed to return to play was also affected by the *ACTN3* R577X genotype. While RR and RX players returned to normal training and competition in ~17–20 days, XX players needed on average 36 days. Clos et al. (15) found a trend towards a longer time to recovery after moderate muscle injury in players with the XX genotype compared to RR and RX genotypes (i.e., ~41, ~22 and ~33 days, respectively), although statistical significance was not reached. Previous mechanistic studies in the α-actinin-3 deficient (*Actn3* ‘knock-out’, KO) mouse model indicate a change in the expression pattern of two important structural proteins, myotilin and desmin (with a uniform localization across all muscle fibers) compared to wild-type mice [30]. This pattern of localization, together with internalized nuclei—which is also found in the *Actn3* KO mouse—are hallmarks of muscle remodeling and regeneration, suggesting that α-actinin-3 deficiency increases susceptibility to ongoing damage [30]. There is some evidence that *ACTN3* genotype might alter the susceptibility to—and progression of—muscle disease, with the X-allele increasing the risk of developing idiopathic inflammatory myopathies [31]. In this context, there is some rationale to support a potential increasing effect of α-actinin-3 deficiency on muscle catabolism after a soccer game [24]. Higher serum levels of indirect markers of muscle damage (creatine kinase, myoglobin) have indeed been reported in athletes with the X-allele compared to the RR genotype after a marathon [19] or half-ironman [20], irrespective of racing performance time. Taken together, the aforementioned information provides further support to the suggested protective mechanical role played by this protein as a scaffold at the sarcomeric Z line [23]. 

The main limitation of the present study is the low sample size and as such, the present findings must be considered preliminary in essence. In turn, a main strength is the unique competition level of the participants together with the homogeneous protocol for muscle injury diagnosis and management that was consistently applied during the study period. Further research in larger cohorts is needed to replicate our findings.

## 5. Conclusions

In summary, we have provided suggestive evidence for the notion that the XX genotype might be potentially associated not only with a higher risk of non-contact muscle injuries, but also with a longer recovery time from these conditions. However, the present findings can only be considered as preliminary in light of the very small cohort we studied. Further research might determine if the XX genotype is really a ‘risk’ condition to be kept in mind by the medical and rehabilitation staff of professional soccer teams. In this regard, the evidence is certainly too initial to support the use *ACTN3* genotyping as a screening tool for prediction of sport-related muscle injuries.

## Figures and Tables

**Table 1:** Main characteristics of the participants by α-actinin-3 gene (*ACTN3*) R577X genotype.

Variable (Units)	RR	RX	XX	*p* Value
Number (frequency, %)	19 (41.3)	22 (47.8)	5 (10.9)	-
Sex (n, % men)	11 (57.9)	9 (40.9)	2 (40.0)	0.518
Age (years, mean ± SD)	25.4 ± 4.1	26.4 ± 5.4	27.8 ± 2.9	0.510
≤25 years (%)	47.4	45.5	20.0	0.529
Height (m, mean ± SD)	1.76 ± 0.07	1.74 ± 0.09	1.74 ± 0.04	0.726
Body mass (kg, mean ± SD)	70.5 ± 9.0	66.8 ± 11.1	66.6 ± 6.6	0.738
BMI (kg·m^−2^, mean ± SD)	22.6 ± 1.7	22.9 ± 1.9	22.0 ± 1.3	0.580
Ethnic origin				0.169
Caucasian (%)	15 (78.9)	21 (95.5)	5 (100.0)	
Afro-American (%)	4 (21.1)	1 (4.5)	0 (0.0)	
Position				0.847
Goalkeeper (%)	2 (10.5)	3 (13.6)	0 (0.0)	
Defender (%)	4 (21.1)	5 (22.7)	2 (40.0)	
Midfielder (%)	8 (42.1)	6 (27.3)	1 (20.0)	
Forward (%)	5 (26.3)	8 (36.4)	2 (40.0)	

Abbreviations: BMI, body mass index.

**Table 2:** Muscle injuries during a 5-year period and α-actinin-3 (*ACTN3*) R577X genotype.

Variable (Units)	RR	RX	XX	*p* Value
Athletes with 0 injuries (n, %)	6 (31.6)	2 (9.1)	0 (0.0)	0.092
Athletes with 1+ injuries (n, %)	13 (68.4)	20 (90.9)	5 (100.0)	
1 injury (n, %)	4 (30.8)	8 (40.0)	3 (60.0)	0.799
2 injuries (n, %)	4 (30.8)	4 (20.0)	1 (20.0)	
≥3 injuries (n, %)	5 (38.5)	8 (40.0)	1 (20.0)	
Return-to-play (days, mean ± SD)	20 ± 10	17 ± 12	36 ± 26 *	0.044

* *p* < 0.05 vs. RX.

## Data Availability

The data presented in this study are available on request from first author of this study.
